# Pituitary function in patients with primary and secondary empty sella

**DOI:** 10.3389/fendo.2025.1632824

**Published:** 2025-07-16

**Authors:** Lucas Steckel, Elke R. Gizewski, Susanne Kaser

**Affiliations:** ^1^ Department of Internal Medicine I, Medical University of Innsbruck, Innsbruck, Austria; ^2^ Department of Radiology, Medical University of Innsbruck, Innsbruck, Austria; ^3^ Neuroimaging Research Core Facility, Medical University of Innsbruck, Innsbruck, Austria

**Keywords:** primary empty sella, hypopituitarism, clinical course, endocrine assessments, secondary empty sella

## Abstract

**Background:**

Due to the increasing availability and sensitivity of neuroradiological imaging, the number of incidental findings of empty sella (ES) is rising, however, the clinical relevance is not clearly defined.

**Methods:**

In this longitudinal, single-center study patients with first-time diagnosed primary or secondary empty sella were analyzed and followed up for five years. Hormone deficiencies were diagnosed by measuring basal pituitary and target organ hormone levels or dynamic stimulation tests.

**Results:**

Overall, 119 patients, 97 with primary (PES) and 22 with secondary empty sella (SES) were included. At baseline, isolated or total pituitary insufficiency was detected in 34% of patients with PES and 63.6% of patients with SES. While hypogonadism was the most common finding in PES affecting 25.8% of patients, adrenal insufficiency was the most frequent finding in SES affecting 54.5% of patients. Only two patients with intact pituitary function at baseline, one with SES and one with PES, were diagnosed with hormone insufficiency during follow-up.

**Conclusions:**

Hormone deficiency is common in empty sella, with males and patients with SES being at highest risk. In patients with intact pituitary function at time of diagnosis, the risk of developing hypopituitarism is low thus not justifying regular follow-up assessments.

## Introduction

Easier access, greater availability and increased sensitivity of magnetic resonance imaging have contributed to strongly increasing findings of empty sella. Neuroimaging finding of empty sella might be incidental but might also be associated with symptoms or hormonal disturbances (1–3).

Empty sella is defined as a herniation of the subarachnoid space into the sella turcica (4). Due to the filling of the space with cerebrospinal fluid the pituitary tissue gets compressed at the bottom and consequently the sella turcica appears empty on neuroradiological imaging (4). The etiology of primary empty sella (PES) is not fully understood, but congenital factors such as diaphragm malformation as well as increased cerebrospinal fluid pressure and variations in pituitary gland volume are discussed to contribute to development of empty sella (5). In contrast, secondary empty sella (SES) results from damage to the pituitary gland such as surgical removal of a pituitary tumor, Sheehan syndrome, traumatic brain injury or radiation therapy, respectively (6).

Data on prevalence of primary empty sella diverge widely from 5.5% to 35% (7–9). The clinical presentation of empty sella is very heterogeneous encompassing asymptomatic patients, patients presenting with neurological symptoms and those with partial or complete pituitary deficiency (1, 2). While recent retrospective or observational studies suggest that anterior pituitary deficiency is common in patients with primary empty sella (1–3, 5, 10), evaluation and management of patients with empty sella are still very heterogenous. In a recent work, Ekhzaimy et al. (11) found that only 20% of patients with incidental neuroimaging finding of empty sella were referred to an endocrinologist.

In this work we aimed to assess the frequency and type of endocrine disturbances in patients with primary or secondary empty sella and the course of disease from an endocrine perspective.

## Materials and methods

### Study population

Patients diagnosed with and treated for empty sella between 2013 and 2023 at the endocrine outpatient clinic at the Department of Internal Medicine I, Medical University Innsbruck were reviewed for participation. Patients with fully available clinical and hormonal data at baseline were enrolled in the study. In some patients only MR reports but not original imaging were available. Analyses included demographic characteristics, symptoms, neuroimaging data and basal hormone levels (prolactin, TSH, fT4, fT3, IGF-1, growth hormone, ACTH, cortisol, FSH, LH, estrogen/testosterone, serum sodium levels) and urinary 24h cortisol levels. GH insufficiency was tested in patients with low or reduced age-adjusted IGF-1 levels or specific symptoms by performing an GHRH arginine test (GHRH: 1 ug/kg body weight, Arginine hydrochloride 0,5 g/kg body weight, maximal dose: 30 g). Stimulated growth hormone (GH) levels lower than 4.1 ng/ml were interpreted as GH deficiency (12).

In patients with specific symptoms of adrenal insufficiency or low basal ACTH and cortisol levels, a standard high dose ACTH stimulation test was performed. Diagnosis of adrenal insufficiency was excluded with peak cortisol levels ≥ 18 ug/dl (500 nmol/l) (13–17).

Hypogonadotropic hypogonadism was diagnosed in male patients with clinical signs, low free testosterone levels and low FSH and LH levels (18). Female hypogonadotropic hypogonadism in women at premenopausal age was diagnosed by amenorrhea for three months or longer, persistently low estrogen levels and low FSH and LH levels. Gonadotropic insufficiency in women at postmenopausal age was diagnosed by low FSH and LH levels (19).

In patients with polydipsia and polyuria, and high or increased serum sodium levels in combination with decreased urine osmolality, a water restriction test followed by nasal insufflation of desmopressin was performed. Doubling of urine osmolality or an increase of urine osmolality by at least 9%/50% to a value > 300 mosmol/kg or > 800 mosmol/kg was interpreted as partial or total arginine desmopressin insufficiency (20–23).

The study protocol was approved by the local ethics committee of the Medical University of Innsbruck (1344/2023).

### Statistical analysis

Non-parametric tests were used for group comparisons (Chi-square test, Fisher’s exact test and Mann-Whitney-U test). The analysis was conducted using the software R (version 4.3.3) and SPSS 29. A p-value ≤ 0.05 was considered as statistically significant. Descriptive data are shown as means +/- SD.

## Results

119 patients, 63 women and 56 men, with radiologic findings of total or partial empty sella were included in the study.

PES was diagnosed in 97 patients and SES in 22 patients. SES was associated with a history of traumatic brain injury in 1 patient, prolactinoma in 3 patients, non-functioning pituitary adenoma in 16 patients and craniopharyngioma in 2 patients.

Baseline characteristics are shown in **Table 1**. An empty sella was diagnosed as cause of specific symptoms in 47 (39.5%) patients with higher rates in patients with PES than in SES (PES: n=40 (41.2%); SES: n=7 (31.8%)). Seventy-two patients (60.5%) did not show any symptoms that are considered as typical or at least indicative of hormonal insufficiencies or an empty sella (7). In patients which were classified as being asymptomatic with respect to the empty sella finding migraine workup and visual disturbances were the most common indications for MR imaging.

**Table 1:** Baseline characteristics of study patients. Nominal variables are denoted in percent (%), continuous data as mean ± standard deviation.

	Total cohort (n = 119)	Patients with primary empty sella (n = 97)	Patients with secondary empty sella (n = 22)
Age (years)	57.7 ± 16.1	57.1 ± 16.1	60.6 ± 16.3
Sex (female/male) (n)	63/56	52/45	11/11
BMI (kg/m^2^)	27.6 ± 6.1	27.2 ± 6.2	29.8 ± 5.6
Symptoms at diagnosis, n (%)	47 (39.5%)	40 (41.2%)	7 (31.8%)
Anterior pituitary deficiency at diagnosis, n (%)	47 (39.5%)	33 (34%)	14 (63.6%)
Hyperprolactinemia at diagnosis, n (%)	21 (17.6%)	17 (17.5%)	4 (18.2%)

Weakness and fatigue were the most frequently found symptoms affecting 25 (21%) and 23 (19.3%) of patients. Weight loss was reported in 6 (5%) cases and galactorrhea in 4 (3.4%) patients. Muscle pain, amenorrhea, reduced libido, skin lesions, polydipsia and polyuria were reported in two patients each (1.7%) and collapse in one patient (0.84%). No gender-specific differences in symptoms were found.

**Figure 1:**
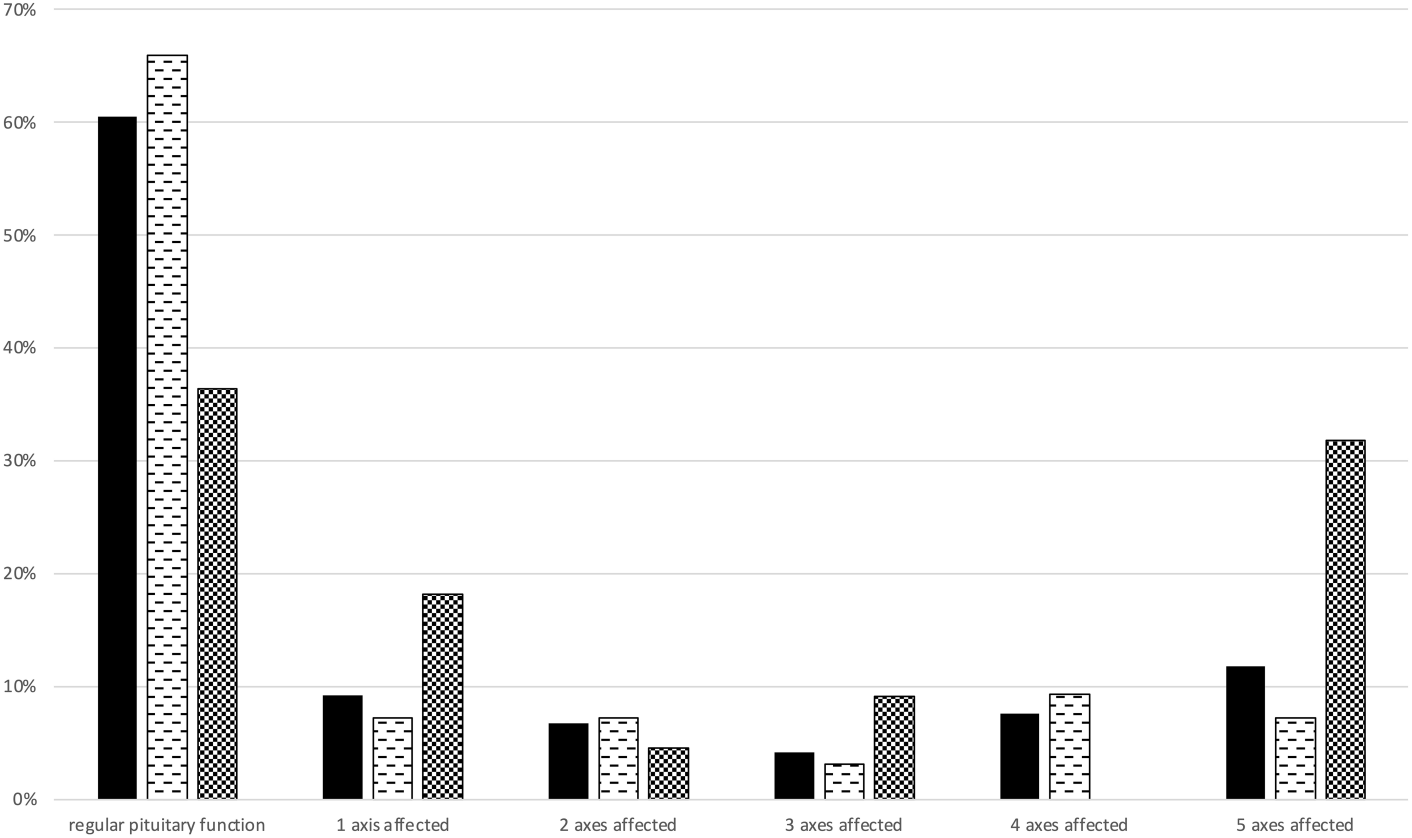
Anterior pituitary function at baseline. Number of patients (in %) with regular pituitary function or 1, 2, 3, 4 or 5 insufficient axes are shown. 1^st^ column (black) indicates the total cohort, 2^nd^ column (dashed) indicates the primary empty sella cohort, 3^rd^ column (checkered) indicates the secondary empty sella cohort.

At baseline total or partial anterior hypopituitarism was diagnosed in 33 (34%) patients with PES and 14 (63.6%) patients with SES. 14 (11.8%) patients suffered from total anterior hypopituitarism, 7 (7.2%) of them belonged to the PES group and 7 (31.8%) to the SES group. The number of affected axes in patients with anterior pituitary deficiency is shown in **Figure 1**. Arginine vasopressin deficiency was diagnosed in 3 (3.1%) patients with PES and 4 (18.2%) patients with SES. Total pituitary insufficiency was found in 3 (2.5%) patients, all of them belonged to the SES group (13.6%). Hyperprolactinemia was found in 17 (17.5%) patients with PES and 4 (18.2%) with SES.

**Figure 2:**
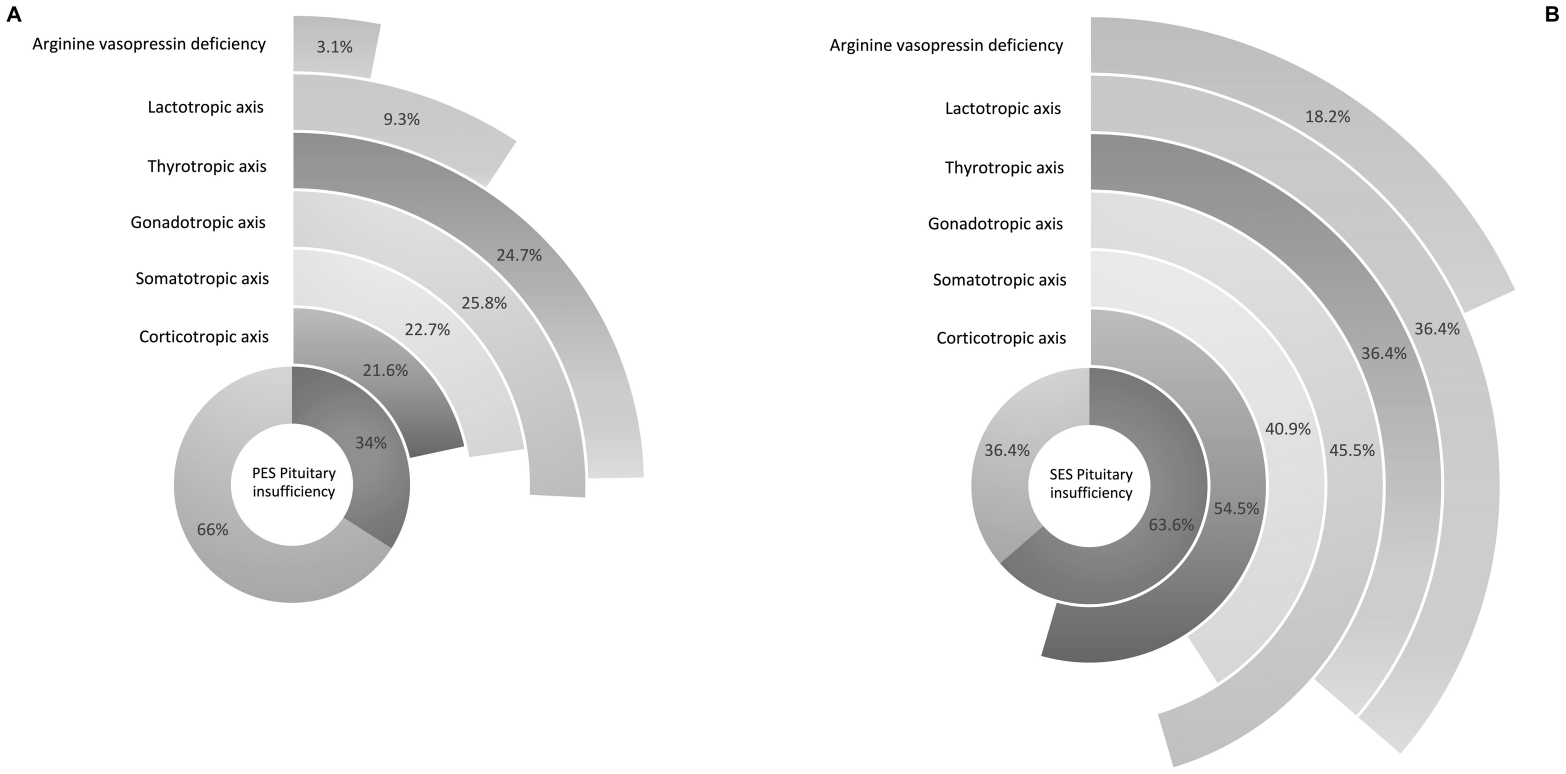
Hormonal insufficiencies shown in percentages of affected patients with primary empty sella (PES) **(A)** and secondary empty sella (SES) **(B)**.

Rates and types of hormonal deficiencies are shown in **Figure 2**.

In patients with PES, hypogonadism was the most frequent finding affecting 25 patients (25.8%) followed by deficiencies of the thyrotropic axis found in 24 patients (24.7%) and the somatotropic axis affecting 22 (22.7%) patients. Adrenal insufficiency was found in 21 (21.6%) patients with PES.

In contrast, 14 (63.6%) patients with secondary empty sella suffered from pituitary insufficiency. Among patients with SES, adrenal insufficiency was the most frequently found hormonal deficiency affecting 12 (54.5%) patients. This was followed by hypogonadism and insufficiency of the somatotropic axis which was found in 10 (45.5%) and 9 (40.9%) patients. Thyrotropic and lactotropic axis were affected in 8 (36.4%) cases each.

Arginine vasopressin deficiency was diagnosed in 4 (18.2%) SES patients compared to 3 (3.1%) PES patients.

In total, the neuroimaging diagnosis was finally classified as an incidental finding in 64 (53.8%).

Pituitary deficiency was more common in males than in females affecting 29 (51.7%) men and 12 women (19.0%), p < 0.001) at baseline. Age, BMI and preexisting endocrinological disorders were comparable in patients with and without pituitary dysfunction at baseline.

**Figure 3:**
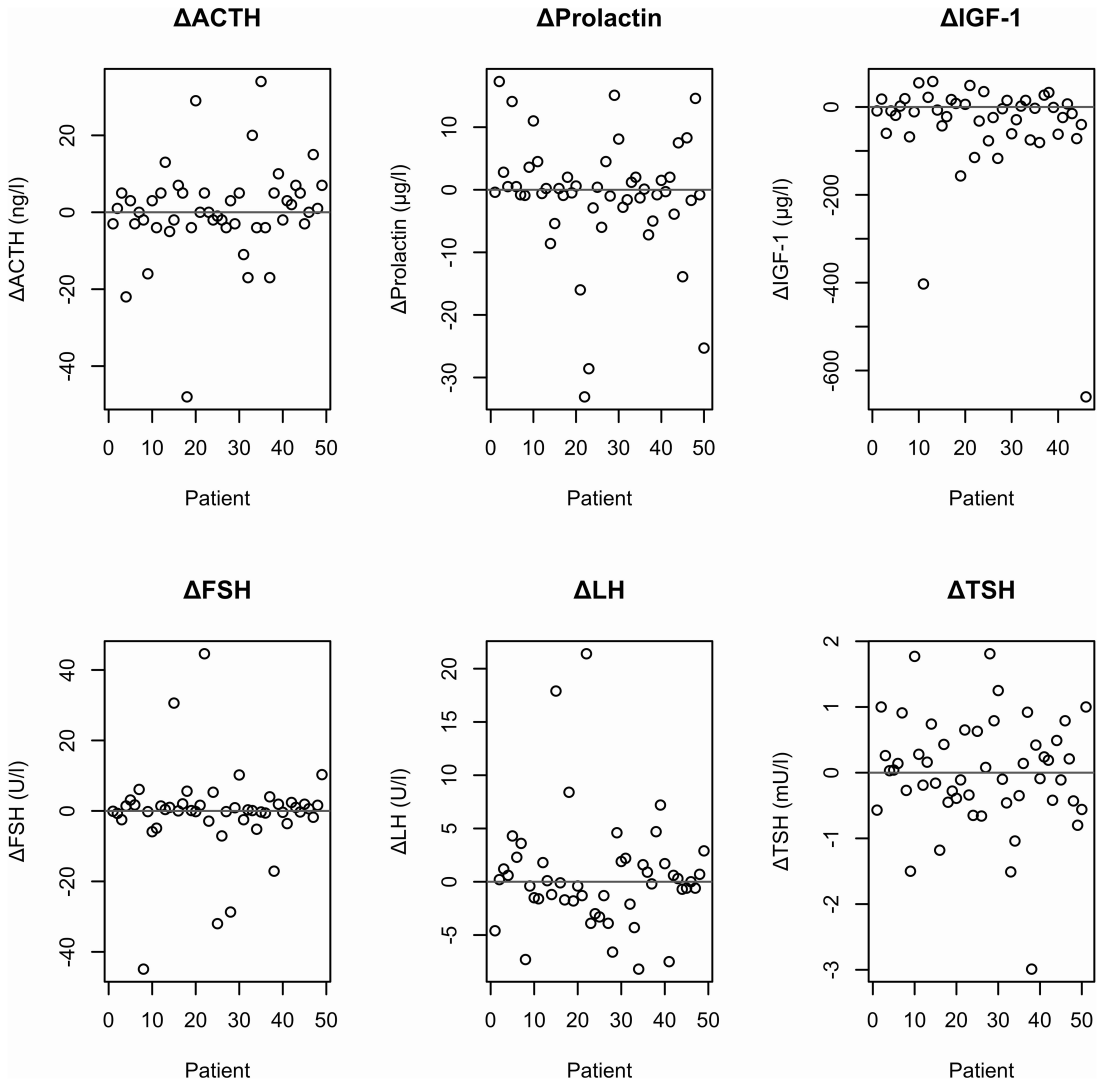
Absolute changes in selected hormone parameters between first presentation and 5-year follow up in patients with intact pituitary function at baseline.

In patients with intact pituitary function at baseline, mean basal hormone levels remained stable over a time period of at least five years indicating stable pituitary function in the majority of patients. Stronger variations were found for FSH and TSH levels only, which might be explained by large physiological and circadian variations of these parameters (**Figure 3**).

To study the course of pituitary function in patients with empty sella, hormonal status was reassessed at least five years after diagnosis in 52 with fully available follow up data and intact pituitary function at baseline. In the primary empty sella cohort, one 69-year-old woman (BMI 24.3 kg/m^2^) with intact pituitary function at baseline developed corticotropic, somatotropic and gonadotropic (defined by low FSH and LH levels) deficiency during follow-up. Function of the thyrotropic axis was not assessed in this patient as she had been diagnosed with and treated for primary hypothyroidism before. Additionally, the patient had been diagnosed with primary hyperparathyroidism before. At initial presentation she suffered from fatigue, however, the symptom was not related with any hormonal deficiency at baseline.

In the secondary empty sella cohort, one patient, a 63-year-old obese woman (BMI 33.7 kg/m^2^) developed deficiencies of the somatotropic and thyreotropic axes during the five-year follow-up. The patient was classified as being asymptomatic at baseline.

## Discussion

Empty sella has become a frequent neuroimaging finding that might be an incidental finding without any clinical relevance but which also might be associated with neurological symptoms or hormone deficiencies. In daily practice, evaluation and management of empty sella findings are very heterogenous and often depend on whether patients are referred to endocrinologists or are treated by GPs. In a recent singly-center study in Saudi Arabia, only 1 of 5 patients were referred to endocrinologists (11).

Data on prevalence of hormonal insufficiencies of disease are limited. In a multi-center study by Carosi et al. (5) investigating patients with primary empty sella, hypopituitarism was found in more than 40%, with hypogonadism being the most frequent finding. In this cohort, hormonal deterioration was uncommon during a mean follow-up of 58 months (5). Importantly, pituitary volume did not predict hypopituitarism in patients with primary empty sella in another study (24). Unfortunately, original imaging was not available from all patients in our study. Accordingly, as a limitation of our study, no measurements of pituitary volume are provided.

The aim of our study was to analyze pituitary function in patients with primary or secondary empty sella and to investigate the natural course of pituitary function over a time period of five years. Importantly, we found that 39.5% of patients with empty sella display partial or total pituitary insufficiency. These results are in line with data from others (1, 3, 5–7, 25–27) including the multicenter study by Carosi et al., who reported that 40% of patients with primary empty sella (including patients with a history of traumatic brain injury) suffered from hypopituitarism (5).

In our study, risk of pituitary insufficiency was markedly higher in patients with secondary empty sella than in those with primary empty sella.

Prevalence of arginine-vasopressin deficiency was low in patients with primary empty sella but affected 4 of 22 patients with secondary empty sella in our study. In total, number of affected axes was distributed evenly. Total anterior hypopituitarism was present in 11.8% of patients. In 76.6% of patients with pituitary insufficiency, at least two hormonal axes were affected.

In patients with primary empty sella and overt anterior pituitary deficiency, hypogonadism was the most frequent finding. Thyrotropic, somatotropic and corticotropic axes were also affected in 21.6% - 24.7% of patients, respectively. Very importantly, in patients with secondary empty sella, adrenal insufficiency was detected in more than 50% of affected patients. Rates of hyperprolactinemia were comparable in patients with primary and secondary empty sella syndrome and was uncommonly associated with presence of galactorrhea.

While the exact pathophysiological mechanism remains to be determined, we hypothesize that mild hyperprolactinemia found in these patients indicate or result from pituitary stalk compression due to filling of the space with cerebrospinal fluid.

These results underline the urgent need of an initial endocrinological assessment in all patients diagnosed with empty sella. Very importantly, prevalence of potentially life-threatening adrenal insufficiency is high, especially in patients with secondary empty sella suggesting the importance of clinical and laboratory evaluation in patients with unspecific symptoms such as fatigue or weakness also.

While empty sella was more prevalent in women (3, 7, 28), pituitary deficiency was more frequent in males than in females in our study suggesting sex-specific differences in etiology and pathophysiology of empty sella syndrome.

Little is known about the natural course of pituitary function in patients with empty sella (5, 29). In our study we found stable basal hormone parameters in the majority of patients. Variations in TSH and FSH levels might rather be explained by natural fluctuations than by changes in pituitary function in patients with empty sella.

Importantly, only two patients, one with primary empty sella and one with secondary empty sella developed pituitary insufficiency over the follow-up period of at least five years, suggesting that aggravation of pituitary function in patients with intact function at diagnosis is rare.

In conclusion, our study shows that pituitary deficiency is common among patients with empty sella and especially frequently found in patients with secondary empty sella. This underlines the urgent need of clinical and endocrinological assessments at diagnosis. Adrenal insufficiency is common, endocrine examination should also include dynamic testing in symptomatic patients. Furthermore, our data suggest against regular follow-up investigations in asymptomatic patients with intact pituitary function at baseline.

## Data Availability

The original contributions presented in the study are included in the article/Supplementary Material. Further inquiries can be directed to the corresponding author.
